# An Inclusive Early Childhood Intervention Program for Children With Disabilities: Possible Effects on Children and Nursery Teachers

**DOI:** 10.3389/fresc.2021.759932

**Published:** 2021-10-14

**Authors:** Kimiko Ueda, Aya Goto, Toshikazu Imamoto, Yoshihisa Yamazaki

**Affiliations:** ^1^Faculty of Health and Well-being, Kansai University, Sakai, Japan; ^2^Center for Integrated Science and Humanities, Fukushima Medical University, Fukushima, Japan; ^3^Department of Psychology, Aichi Gakuin University, Nisshin, Japan; ^4^Aichi Children's Health and Medical Center, Obu, Japan

**Keywords:** inclusive early childhood intervention, children with disabilities, developmental quotient, self-efficacy, anxiety

## Abstract

Inclusive early childhood intervention provides opportunities for children with disabilities to receive education with typically developing children. The present study examined the effects of the AI-AI STEP Program, which is designed to help nursery teachers learn the methods of inclusive early childhood intervention for children with disabilities. This study involved 37 managers of 37 nursery schools in Japan, 48 nursery teachers, and 48 children with disabilities. The school managers, who had previously learned about the program through a seminar we offered, provided the nursery teachers with guidance on the program. The guidance provided to the nursery teachers consisted of combined structured explanations with a manual and on-the-job training. The program was performed for 6 months, and changes in the children's development and behavior and the school nursery teachers' self-efficacy and state-trait anxiety, were examined before and after using the program. Multivariate analysis was used to assess factors that had an effect on the children's developmental gains through the program. The developmental quotient of children significantly improved. In addition, “emotional symptoms” and “peer problems” on the Strengths and Difficulties Questionnaire subscale markedly improved. The self-efficacy of nursery teachers significantly improved, and state anxiety decreased. There was a significant relationship between the improvement of the children's development quotient and a lower development quotient at baseline. The provision of inclusive early childhood intervention using the program promoted the children's development, and improved their behavior. Furthermore, it had a positive effect on the nursery teachers.

## Introduction

Early childhood intervention (ECI) is a provision of support for children with disabilities according to their developmental stages. ECI is one of a variety of evidences suggesting that early childhood intervention provides an important opportunity to alter children's development and children-parent interactions (1–3). Children with disabilities receiving education with typically developing children is an important aspect of inclusive early childhood intervention (IECI) (4–6). Communication among the children through daily life and play activities has a positive effect. Lindsay (7) and Rafferty et al. (8) reported that IECI promoted the language development of preschoolers with disabilities and improved their social adaptation ability. It also enhanced typically developing children's sociability and increased the numbers of their friends (9).

In Japan, IEI is defined as “education and care for infants with physical and/or intellectual disabilities provided in nursery schools.” In 1974, the Ministry of Health and Welfare (currently, the Ministry of Health, Labor, and Welfare) launched a project for children with disabilities to receive education and care in nursery schools. It was an innovative attempt to incorporate nursery schools, in addition to rehabilitation centers, into Japan's IECI system for children with disabilities. Currently, 92.7% of all Japanese nursery schools provide IECI for such children (10). These schools are offered financial support to increase the number of nursery teachers or adopt other necessary measures; however, some nursery schools have insufficient financial resources, staff, and quality assurance.

IECI for children with disabilities in nursery schools is supported by experts through two types of service: on-site counseling and nursery school-visit services. The former is operated by municipalities that dispatch doctors, psychologists, or other experts to nursery schools, and provide guidance for nursery teachers. These experts directly advise nursery teachers about how to provide childcare to children with disabilities. In the latter, which started in April 2012, experts from the child developmental support centers visit nursery and elementary schools in communities to directly provide specialized support for children with disabilities.

The Japanese government has recently begun to incorporate IECI into its general policies for children with or without disabilities. In April 2015, it launched the Comprehensive Support System for Children and Childcare by three central government entities: the Cabinet Office; the Ministry of Education, Culture, Sports, Science and Technology; and the Ministry of Health, Labor and Welfare. Importance was attached to close collaboration to simultaneously support children with disabilities and their parents. It is necessary to further organize systems to provide IECI for children with disabilities in nursery schools, and improve their quality; however, there are limited assessments of IECI and evaluation of its impact on children in Japan.

Since 2004, the Aichi Children's Health and Medical Center has been training leaders of nursery teachers using the AI-AI STEP (Step TEaching Program) Program (11). The first “AI” represents the Japanese “word for love/ attachment” and the other “AI” is and abbreviation of “Aichi”, the birthplace of the program. The program is designed to help nursery teachers learn the methods of IECI for target preschool children aged 2–6. Target children include those with suspected autism spectrum disorder, attention deficit hyperactivity disorder, learning disability, or other developmental disorders. When the program started, the recognition of developmental disorders was insufficient in Japan. In a large number of such cases, if the disorder had been identified and appropriately managed earlier, the situation may have been less stressful for both the child and the people around him/her. Many nursery teachers sought advice on individualized approaches from the experts of the Aichi Children's Health and Medical Center. As a result, the center began to provide opportunities for nursery teachers to learn about IECI, with the aim of enabling children with disability to perform group activities more comfortably at nursery schools, while enhancing nursery teachers' self-confidence in providing IECI. To date, more than 600 nursery teachers have been trained at the center using this program.

We examined the effects on target children and nursery teachers of the AI-AI STEP Program, and assessed factors that had an effect on the children's developmental gains through the program.

## Materials and Methods

### Contents of the AI-AI STEP Program

The AI-AI STEP Program aims to support children's appropriate behaviors, focusing on their relationships with nursery teachers (11). The AI-AI STEP Program is a practice-based program based on behavioral science (12, 13) and social learning theory (14, 15). It is characterized by four points: (1) being useful for all nursery teachers; (2) being useful for all children, regardless of the characteristics of their disability; (3) facilitating practical verification; and (4) attaching importance to experience-based learning. The program integrates individualized and grouping approaches.

The individualized approaches correspond to a target child's three behavioral stages: (1) behaviors to be developed (to perform self-care), (2) behaviors to be adopted (to overcome difficulties or acquire new experiences), and (3) behaviors to be avoided (problematic or possibly problematic behaviors). The nursery teachers focus on these behaviors and select a behavior from stage 1 that they consider to be easier to correct. The process has six steps: (1) observing behaviors, (2) setting three support processes, (3) practicing (making commitments), (4) recording, (5) assessing (grading), and (6) summarizing. When the target child can perform the behavior properly, the nursery chooses a behavior from stage 2 and supports the child using the same six steps. A nursery teacher starts with an easier task (stage 1), and proceeds to the more difficult tasks (stage 2 or 3). These three stages and six steps enable nursery teachers to acquire and improve their objective observation, practical, and verification skills.

The grouping approaches are based on the idea that groups can be appropriately created by establishing favorable relationships between target children and nursery teachers, between target and other children, and between other children and nursery teachers, rather than by simply including target children in groups. Nursery teachers' behaviors are an exemplary for children at all times. The use of affirmations for target children by nursery teachers, such as praising them in front of other children, promotes the latter's affirmative behaviors toward the former. Such approaches are effective for other children to observe and develop appropriate attitudes toward the target children. They also promote affirmative behaviors and attitudes, such as ‘praising', among all class members.

### Targets and Study Design

The present study was a pre-post design quasi-experimental study. This study consisted of 37 managers of 37 nursery schools in the Aichi and Fukushima Prefectures, Japan; 48 nursery teachers; and 48 children with disabilities. The school managers, who had previously learned about the program through an intensive seminar we offered, provided the nursery teachers in charge of the children with disabilities with guidance on the program at their nursery schools. The managers' guidance consisted of combined structured explanations with a manual and on-the-job training. Subsequently, based on the program and with support from the school managers, the nursery teachers provided IECI for the children with disabilities for 6 months. Fidelity assessments of nursery teachers' IECI were conducted by managers using direct observation and reviews of the nursery teachers' self-reports that were completed in steps 4–6. Before and after IECI using the program, changes in the children's development and behaviors, as well as those in the school nursery teachers' self-efficacy and state-trait anxiety, were examined.

## Measures

The children's development and behavior were assessed using the Kinder Infant Development Scale (KIDS) (16) and Strengths and Difficulties Questionnaire (SDQ) (17, 18), respectively, both of which are self-administered, registered questionnaires, which were filled out by the nursery teachers. The nursery teachers' self-efficacy and state-trait anxiety were evaluated using the General Self-Efficacy Scale (GSES) (19) and a new version of the State-Trait Anxiety Inventory (STAI) (20), respectively.

### Kinder Infant Development Scale (KIDS)

KIDS is a parent/caregiver-rated questionnaire for developmental screening of children aged 1 month to 6 years and 11 months that has been used since 1989 all over Japan (16). There are four types of questionnaires for children of different ages. We used Type T, which is recommended for children aged 1 month to 6 years and 11 months with developmental delay. The questionnaire is composed of nine subscales with 282 items: Physical-Motor (37 items), Manipulation (37 items), Receptive Language (37 items), Expression Language (37 items), Language Concepts (25 items), Social Relationships with Children (25 items), Social Relationships with Adults (37 items), Discipline (25 items), and Feeding (22 items). For each item, the nursery teacher answered with a circle if the child could perform the behavior and a cross if the child could not. The raw score for the subscale was the number of circles, which were converted into developmental age (DA) by referring to a translation table in the KIDS manual. Development quota was calculated by dividing DA by the child's chronological age.

### Strengths and Difficulties Questionnaire (SDQ)

We used the Japanese version of SDQ (21). The SDQ is a brief screening instrument to assess the positive and negative aspects of the behaviors of children and adolescents assessed by parents and teachers. The SDQ consists of five subscales with 25 items, four kinds of problem scores (emotional symptoms, conduct problems, hyperactivity/inattention, and peer problems), and a positive aspect of prosocial behavior. The SDQ had three item responses: 0 = not true, 1 = somewhat true, and 2 = certainly true. Higher scores on the four subscales reflect behavioral difficulties, whereas higher scores on the prosocial behavior subscale reflect strengths.

### The General Self-Efficacy Scale (GSES)

The GSES was developed by Sakano and Tohjoh (19). It is an instrument for measuring an individual's strength of general self-efficacy across a variety of everyday life settings. Bandura (22), who originally proposed the concept, defined self-efficacy as a judgment of “how well-one can execute courses of action required to deal with prospective situations.” The total score is calculated by the sum of all 16 items. We used standardized scores based on a conversion table indicating 50 average points and a standard deviation of 10. A higher score means more self-efficacy.

### The State-Trait Anxiety Inventory (STAI)

STAI, which was developed by Spielberger et al. (20), is a psychological inventory based on a 4-point Likert scale and consists of 40 questions on a self-report basis. It measures two types of anxiety: state anxiety and trait anxiety. Each type of anxiety has its own scale of 20 different questions scored from 20 to 80. Higher scores indicate greater anxiety.

## Data Analysis

Changes in these items after intervention with the AI-AI STEP Program were examined using a paired *t*-test. Furthermore, multivariate analysis was used to assess factors that had an effect on the children's developmental gains through the AI-AI STEP Program. The number of children was small; therefore, in the multivariate analysis, the children's and the nursery teachers' factors were selected based on correlation coefficients of associations between each factor and the children's developmental gains.

## Ethical Consideration

We explained the study protocol to the school managers and the nursery teachers, then informed the parents of the children. The study was conducted with written informed consent from the school managers, the nursery teachers, and the parents of the children, and the approval of the ethics committee of the institution the first author belonged to (No. 599).

## Results

Table 1 shows the characteristics of the target children and nursery teachers. The mean ages of the school managers, nursery teachers, and children were 56, 37, and 5 years and 9 months, respectively. The number of children in each group was as follows: undiagnosed but suspected disorders (*n* = 26), autism spectrum disorder (*n* = 10), intellectual disorder (*n* = 4), Down syndrome (*n* = 2), and physical disabilities (*n* = 2).

**Table 1:** Characteristics of children and nursery teachers.

	***N*** **(%) or mean (SD)**	
**Children (N** **=** **48)**		
Sex		
Male	37	(77.1)
Female	11	(22.9)
age (mean, SD)	53.8 months	(11.1)
two	4	(8.3)
three	9	(18.7)
four	20	(41.7)
five	12	(25.0)
six	3	(6.3)
diagnosis		
ASD	14	(29.2)
Suspicion of ASD	26	(54.1)
DS	2	(4.2)
ID	4	(8.3)
PD	2	(4.2)
**Nursery teacher (N** **=** **48)**		
sex		
male	2	(4.2)
female	46	(95.8)
age	36.6	(9.7)
**School Manager (N** **=** **37)**		
sex		
female	37	(100.0)
age	56.2	(3.4)

*ASD, autistic spectrum disorder; DS, Down syndrome; ID, Intellectual disability; PD, physical disability*.

Table 2 shows the pre-/post-program scores of the children's KIDS and SDQ, and the nursery teachers' GSES and STAI. After IECI using the AI-AI STEP Program, the developmental quotient significantly improved in the KIDS in all subscales. Furthermore, “emotional symptoms” and “peer problems” on the SDQ subscale markedly improved, regardless of the disorder. On comparing the female nursery teachers before and after the AI-AI STEP Program, GSES-standardized scores significantly improved, with decreases in scores related to state anxiety. The lower state anxiety of nursery teachers had a significant relationship with reduced anxiety (coefficient −0.49, *p* < 0.0005).

**Table 2:** Pre/post scores of children's KIDS and SDQ, and nursery teacher's GSES and STAI.

	**Pre-test scores**		**Post-test scores**		
	**Mean**	**(S.E.)**	**Mean**	**(S.E.)**	***p*-value score**
**Children**					
Kinder infant development scale (KIDS)					
Physical-motor	37.4	(1.9)	43.5	(1.9)	<0.001
Manipulation	37.5	(2.8)	49.8	(2.8)	<0.001
Receptive language	37.6	(2.7)	45.5	(2.6)	<0.001
Expression language	32.7	(2.5)	39.0	(2.4)	<0.001
Language concepts	39.5	(2.6)	45.2	(2.3)	<0.001
Social relationships with children	33.7	(2.3)	39.9	(2.3)	<0.001
Social relationships with adults	28.8	(2.5)	33.8	(2.4)	0.02
Discipline	42.6	(2.2)	48.1	(2.3)	<0.001
Feeding	23.3	(1.6)	27.3	(1.2)	<0.001
Developmental quotient	63.6	(3.6)	70.0	(3.2)	0.01
Strengths and difficulties questionnaire (SDQ)					
prosocial behavior	2.3	(0.4)	2.5	(0.4)	0.24
hyperactivity/inattention	6.5	(0.4)	6.8	(0.3)	0.79
emotional symptoms	2.9	(0.3)	2.5	(0.3)	0.04
peer problems	5.0	(0.3)	4.4	(0.3)	0.04
conduct problems	2.6	(0.3)	2.8	(0.3)	0.80
total difficulties	16.9	(0.8)	16.4	(0.8)	0.20
**Nursery teacher (N=48)**					
General self-efficacy scale (GSES)					
standardized score	42.4	(1.3)	44.1	(1.3)	0.03
male (*N* = 2)	35.5	(4.5)	35.0	(1.0)	
female (*N* = 46)	42.7	(1.3)	44.5	(1.4)	0.03
State-trait anxiety inventory (STAI)					
State anxiety	46.2	(1.2)	42.3	(1.2)	0.001
Trait anxiety	46.6	(1.3)	45.1	(1.2)	0.059

Improvement of the children's development quotient was significantly affected by a lower children's development quotient at baseline, the diagnosis of autism spectrum disorder, and higher state anxiety inventory at baseline of the children's nursery teacher (Table 3).

**Table 3:** Linear regression predicting child's development quotient gain by the program.

**Variables**	**Coefficient**	***p-value***	**95% Confidence interval**	
Child's development quotient at baseline	-0.39	<0.001	-0.56	-0.23
Total difficulties of strengths and difficulties questionnaire	0.41	0.29	-0.37	1.19
The diagnosis of autism spectrum disorder	13.5	0.02	2.2	24.8
State anxiety inventory at baseline	-0.63	0.02	-1.15	-1.01

## Discussion

The present study examined the effects of the AI-AI STEP Program to support IECI for children with disabilities on the children themselves and the nursery teachers in charge of them. The provision of IECI using the program increased the nursery teachers' self-efficacy and decreased anxiety. The nursery teachers had more confidence in their ability to address the needs of children with disabilities after the program. The program also promoted the children's development, and improved their behavior, in particular emotional symptoms and peer problems. The improvement of the children's development was larger for those children who had larger developmental delays or who had a diagnosis of autism spectrum disorder at baseline.

As the children's developmental index values increased even when adjusting for chronological age, it was confirmed that the program promoted children's development. Improvements in children's language development and social adaptation ability were also reported in some previous studies (8, 9). The present study adds to this research by comprehensively examining developmental changes using KIDS. However, because of the pre-post design of our quasi-experimental study, history effects, and the Hawthorne effect may have influenced our results. Further studies that include randomized controlled trials using a control group.

Among the nursery teachers, both state-related anxiety and general self-efficacy scores improved after starting to use the program. Most nursery teachers perceived providing IEI for children with disabilities to be challenging; therefore, the program may have reduced their anxiety through the learning of practical methods for education and care. In addition, the reduction in their state anxiety was more marked when their state anxiety was higher at baseline, suggesting that their anxiety was reduced by having school managers' support and realizing the positive effects of their own approaches on children. In a previous study that taught nursery teachers about health literacy (how to convey health-related messages to the target audience), we observed that participating teachers who used the learned skills continued to have confidence in conveying health messages even 1 year after the training (23). Both projects indicate that acquiring practical skills and their application in the field improve professional confidence.

This study has some limitations. First, the number of subjects was limited. Second, as the design of this study had a one-group pre-post design, it was difficult to clarify whether the effects had been achieved through intervention or as a result of child development. Furthermore, we were unable to ascertain whether the program directly influenced the nursery teachers' self-efficacy using their assessment results or whether other outside factors had an effect. Third, the children's outcomes may be overestimated because of differing perspectives among the teachers. Finally, the mechanism that led both the children and their teachers to change through the program remained unclear. The theoretical background and validation of the program were insufficient because it was a practice-based program. Thus, it may be necessary to use a control group in future studies or conduct randomized control studies with more detailed data collection from the both groups.

## Data Availability Statement

The raw data supporting the conclusions of this article will be made available by the authors, without undue reservation.

## Author Contributions

KU, AG, TI, and YY: material preparation and data collection were performed. KU and AG: analysis was performed. KU: the first draft of the manuscript was written. All authors contributed to the study conception and design, commented on previous version of the manuscript, read, and approved the final manuscript.

## Funding

This study was supported by Research on Region Medical, Health, Labor and Welfare Sciences Research Grants (201232063A) by Japanese Ministry of Health, Labor and Welfare.

## Conflict of Interest

The authors declare that the research was conducted in the absence of any commercial or financial relationships that could be construed as a potential conflict of interest.

## Publisher's Note

All claims expressed in this article are solely those of the authors and do not necessarily represent those of their affiliated organizations, or those of the publisher, the editors and the reviewers. Any product that may be evaluated in this article, or claim that may be made by its manufacturer, is not guaranteed or endorsed by the publisher.
